# Screening of Oral Potential Angiotensin-Converting Enzyme Inhibitory Peptides from *Zizyphus jujuba* Proteins Based on Gastrointestinal Digestion In Vivo

**DOI:** 10.3390/ijms242115848

**Published:** 2023-10-31

**Authors:** Xinchang Gao, Chaoying Zhang, Ning Wang, Jin-Ming Lin, Yali Dang, Yufen Zhao

**Affiliations:** 1 Institute of Drug Discovery Technology, Ningbo University, Ningbo 315211, China; gxch43@126.com (X.G.); wangning2@nbu.edu.cn (N.W.); 2 Department of Chemistry, Tsinghua University, Beijing 100084, China; 3 Chinese Academy of Fishery Sciences, Beijing 100141, China; cyzhang0317@cafs.ac.cn; 4 College of Food and Pharmaceutical Sciences, Ningbo University, Ningbo 315211, China

**Keywords:** *Zizyphus jujuba*, ACE-inhibitory peptides, screening, digestion stability, 3D-QSAR model

## Abstract

Plant proteins are a good source of active peptides, which can exert physiological effects on the body. Predicting the possible activity of plant proteins and obtaining active peptides with oral potential are challenging. In this study, the potential activity of peptides from *Zizyphus jujuba* proteins after in silico simulated gastrointestinal digestion was predicted using the BIOPEP-UWM™ database. The ACE-inhibitory activity needs to be further investigated. The actual peptides in mouse intestines after the oral administration of *Zizyphus jujuba* protein were collected and analyzed, 113 *Zizyphus jujuba* peptides were identified, and 3D-QSAR models of the ACE-inhibitory activity were created and validated using a training set (34 peptides) and a test set (12 peptides). Three peptides, RLPHV, TVKPGL and KALVAP, were screened using the 3D-QSAR model and were found to bind to the active sites of the ACE enzyme, and their IC_50_ values were determined. Their values were 6.01, 3.81, and 17.06 μM, respectively. The in vitro digestion stabilities of the RLPHV, TVKPGL, and KALVAP peptides were 82%, 90%, and 78%. This article provides an integrated method for studying bioactive peptides derived from plant proteins.

## 1. Introduction

*Zizyphus jujuba*, a famous oriental traditional medicine, is rich in nutrients, such as proteins (with a 3–7% dry weight), carbohydrates (including fiber polysaccharides at a 2–9% dry weight), vitamins (especially V_B_ and V_C_), and important minerals (e.g., manganese, iron, phosphorus, potassium, magnesium, calcium, and zinc) [1]. *Zizyphus jujuba* extracts show anti-anaphylactic activity [2], food-borne pathogen inhibition [3], plasmin activity regulation [4], and antioxidant [5] and antibacterial activity [6].

There is a large number of reports on bioactive peptides from plants. Yang et al. [7] reported the preparation of ACE-inhibitory (ACEI) peptides from rice proteins using a multi-mode S-type ultrasound. Sonklin et al. identified antihypertensive peptides in Mung bean protein hydrolysates and their effects on spontaneously hypertensive rats [8]. The plant-derived bioactive peptides inhibited enzymes like alpha-glucosidase, alpha-amylase, dipeptidyl peptidase-IV, and the glucose transporter systems involved in type 2 diabetes [9].

However, there are few reports on *Zizyphus jujuba* proteins and hydrolysates. Two antioxidant peptides, VGQHTR and GWLK, and two ACEI peptides, IER and IGK, respectively, were isolated from the protein hydrolysates of *Zizyphus jujuba* [10,11]. Up to now, there has been a lack of systematic studies on the functions of *Zizyphus jujuba* proteins. 

Peptidomics is a non-targeted method for the rapid identification of protein hydrolysates or crude peptide components. Because this method is simple to operate and does not require the step of separating peptides, it can save a lot of time [12]. The advent of virtual screening and efficient technologies like bioinformatics has highly revolutionized the process of drug design. There are a lot of studies on peptidomics and virtual screening that investigate biological systems. Hou et al. [13] established a peptidomics and bioinformatics method to screen novel antimicrobial peptides from pepper seeds. A total of 16 potential antimicrobial peptides were screened from 4358 peptides via molecular docking. Zhang et al. [14] identified 543 peptides from pea protein via peptidomics and screened 4 novel DPP-IV-inhibitory peptides with an IC_50_ < 100 μM via molecular docking. Yu et al. [15] used bioinformatics and molecular docking to screen 2 ACEI peptides from 1276 peptides derived from ovo-transferrin.

The function of ACEI peptides is related to their structure. Prabhakar and Gupta [16] performed the first basic quantitative structure–activity relationship (QSAR) study on ACE inhibitors. Yan et al. [17] reported that ACEI peptides were affected by many factors, such as their molecular mass, amino acid type, sequence, etc. Most of the currently detected ACEI peptides are short peptides, and the number of amino acid residues is usually 2–12. The side chains of ACEI peptides are large and hydrophobic dipeptides. C-terminal aromatic amino acids and intermediate positively charged amino acid tripeptides had good biological activity [18]. The His383, Glu384, His387, and Glu411 residues of ACE were found to be the main binding sites of ACEI peptides via molecular dynamics and molecular docking [19,20]. In addition, Yan et al. [17] revealed an interaction mechanism that Zn synergizes with ACEI peptides to assist in the nucleophilic attack of activated water molecules, leading to changes in the structure of the active sites, as shown by the 3D-QSAR model. 

Protein hydrolysates may exhibit good ACE-inhibitory activity in vitro since bioactive peptides are degraded and modified in the intestines, vascular system, and liver, and it is important to examine their activity in vivo [21]. It is well known that the gastrointestinal tract is one of the main obstacles in the human body. The gastrointestinal environment, such as the digestive enzymes in the gastrointestinal tract and the pH in the stomach, may affect the structure and function of peptides [22]. Miquel et al. [23] reported the potential mineral-chelating properties of caseinophosphopeptides (CPPs) associated with the presence of amino acid sequences and phosphorylated clusters in the formation and identification of casein phosphopeptides after in vitro simulated physiological digestion. In addition, the effect of in vitro gastrointestinal digestion was demonstrated on the activity of peptides with specific sequences [24]. However, bioactive peptides from natural foods are always composed of thousands of peptides that exert similar properties and potential interactions during gastrointestinal digestion [25]. Accordingly, it is important to isolate and identify peptides that are active in the gastrointestinal environment.

In this paper, bioinformatics methods were used to predict the potential activity of jujube proteins after in silico simulated gastrointestinal digestion, and a 3D-QSAR model was further used to screen and validate the ACE-inhibitory activity of the peptide segments produced in the intestines of mice after the oral administration of jujube proteins. This study provides a new perspective on the utilization of natural-source proteins and the screening of active peptides with potential for oral administration.

## 2. Results and Discussion

### 2.1. Preparation and Components of Zizyphus jujuba Proteins

The components of *Zizyphus jujuba* proteins were analyzed using proteomics. The 15 *Zizyphus jujuba* proteins with the most intensity were studied via the computer simulation of gastrointestinal digestion (Table S1), and the activity of the peptides produced after digestion was predicted.

**Figure 1:**
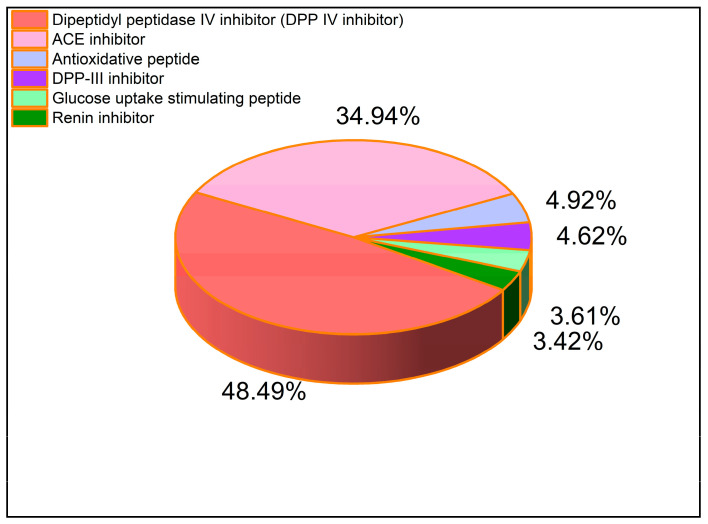
Diagram of the potential activity of *Zizyphus jujuba* proteins after in silico gastrointestinal digestion.

Based on the BIOPEP-UWM database, the function of bioactive peptides was further analyzed, which could be used for the detection of bioactive peptides, including antioxidant peptides, ACE inhibitors, dipeptidyl peptidase IV inhibitors (DPP-IV), dipeptidyl peptidase III inhibitors (DPP-III), and glucose-uptake-stimulating peptides. Using the BIOPEP-UWM database, we predicted the activity of the peptides produced by the *Zizyphus jujuba* proteins after simulated gastrointestinal digestion. Interestingly, among these potential bioactive peptides, we found that potential ACEI peptides accounted for nearly 34.94% of all the peptides isolated from the sample (Figure 1). These potential ACEI peptides may have also contained other potential functional peptides, such as DPP-IV-inhibitory peptides and antioxidative peptides. According to the results of the simulated enzyme digestion analysis, the ACEI activity was selected for further research in this study.

### 2.2. Peptidomics Analysis of Zizyphus jujuba Protein In Vivo 

The results of the peptidomics analysis showed that there were nearly 1000 peptides with an ALC% > 80 in the intestine samples of the mice given *Zizyphus jujuba* proteins, and the common 705 peptides contained in the three samples were collected for the next experiment. Of the 705 co-produced peptides, we removed the peptides present in the gut of the blank mice, and the final total of 113 peptides was used for the ACE-inhibitory activity screen; see Supplementary Materials Table S3.

### 2.3. ACE-Inhibitory Activity of Jujube Protein after In Vitro and In Vivo Digestion

The enzymatic hydrolysates of the jujube protein showed high ACE-inhibitory activity after both in vitro and in vivo digestion. The ACE-inhibitory IC_50_ of the jujube protein after simulated gastrointestinal digestion was 0.34 mg/mL. The ACE-inhibitory IC_50_ of the jujube protein hydrolysates in the intestinal tract of the mice was 0.21 mg/mL. See Table 1.

### 2.4. Setting 3D-QSAR Model

**Figure 2:**
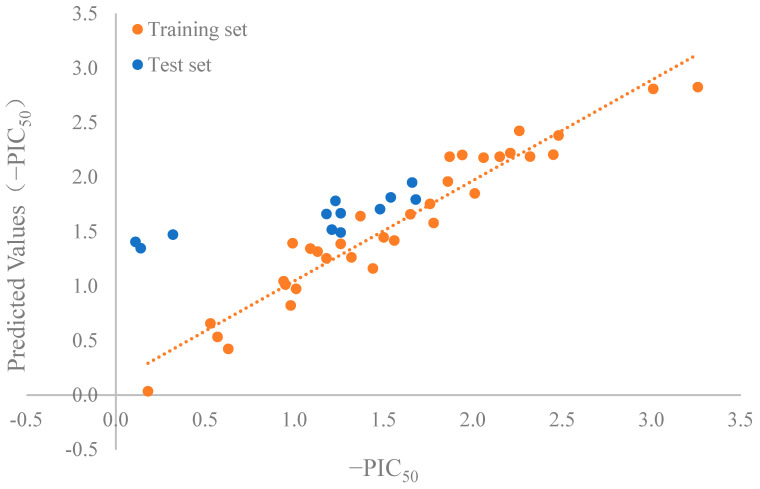
Parameters of 3D-QSAR model. Correlation R^2^ = 0.927, Q^2^ = 0.706.

In the parameters of the 3D-QSAR model, the leave-one-out cross-validated coefficient (Q^2^) was 0.706 and the leave-one-out non-cross-validated coefficient (R^2^) was 0.927 in Figure 2. This indicated that the models were consistent with the recommended reference values of the 3D-QSAR model (Q^2^ > 0.5, and R^2^ > 0.9) [26]. Qi et al. developed a 3D-QSAR model (Q^2^ = 0.773, R^2^ = 0.992) and identified four novel tripeptide inhibitors of ACE [27].

### 2.5. 3D-QSAR Model Prediction Results and Validation

According to the prediction results of the 3D-QSAR model, the 10 ACEI peptides with the highest scores were synthesized for ACE-inhibitory activity verification. The binding of the peptides to ACE was affected by their amino acid sequence and type. Leucine, proline, and phenylalanine could promote the better binding of peptides to ACE, especially when these hydrophobic amino acids appeared in the side chain [28,29]. In Table 2, we see that only KVKPL had no significant inhibitory activity on ACE at the peptide concentration of 2 mg/mL. The other nine peptides all had inhibitory effects on ACE. The RLPHV, TVKPGL, and KALVAP peptides exhibited remarkable inhibitory activity, with a 100% inhibition rate at a concentration of 2 mg/mL. Subsequent experiments revealed the IC50 values of RLPHV, TVKPGL, and KALVAP to be 6.01, 3.81, and 17.06 μM, respectively. Among them, TVKPGL had the lowest IC_50_, and the strong ACEI activity was in line with the rule of strong ACEI active peptides of solid hydrophobic activity; additionally, the C-terminal amino acid was proline or a hydrophobic amino acid. The RLPHV peptide contained a positively charged amino acid (arginine) at the N-terminal, which might be one of the reasons for its strong ACEI activity. The arginine and phenylalanine residues in the RSFCA peptide were reported to be essential for the specific interactions in ACE inhibition [30]. The structure–activity results suggested that the positively charged N-terminal arginine or lysine, in the guanidino or ε-amino groups, respectively, appeared to contribute significantly to the ACE-inhibitory potency [31]. Therefore, there may be an interaction between the inhibitor and an anion-binding site of ACE different from the catalytic site. The C-terminal arginine residue being removed could essentially lead to inactive peptide analogs [32,33].

### 2.6. Molecular Docking

**Figure 3:**
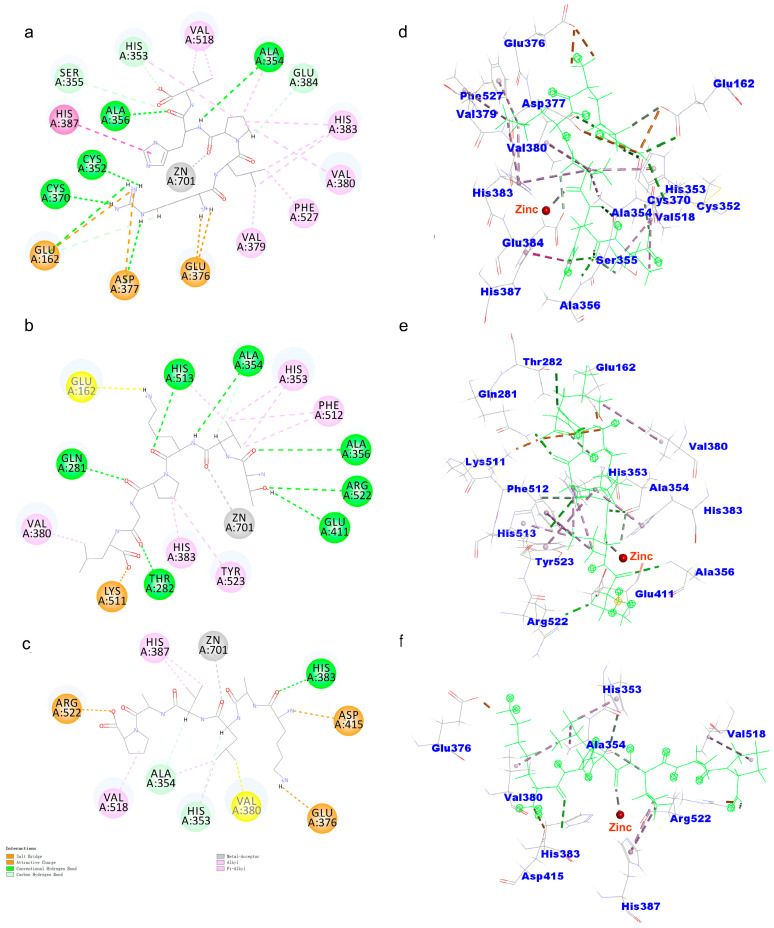
Molecular docking of three peptides: (**a**) 2D diagram of the interaction between RLPHV and ACE, (**b**) 2D diagram of the interaction between TVKPGL and ACE, (**c**) 2D diagram of the interaction between KALVAP and ACE, (**d**) 3D diagram of the interaction between RLPHV and ACE, (**e**) 3D diagram of the interaction between TVKPGL and ACE, and (**f**) 3D diagram of the interaction between KALVAP and ACE.

ACEI peptides were obtained via 3D-QSAR virtual screening, and the dose–effect relationship between the peptides and the ACEI activity was quantitatively determined using the hippuric acid content produced by the ACE hydrolysis substrate Hippuryl-histidyl-leucine (HHL). However, the structure–activity relationship between the peptides and the ACEI activity was not directly shown. Therefore, Discovery Studio 2020 was used to demonstrate the molecular docking mechanism between the peptides and ACE. The binding energy of the peptides to ACE is shown in Table 3. The different binding affinities and binding modes stabilized the ACE–peptide complex in Figure 3. The binding energy of the *Zizyphus jujuba* peptides to ACE ranged from −106.075 to −102.312 kcal/mol. RLPHV bound to the sites of ACE (Cys352, His353, Ala354, Ser355, Ala356, Cys370, Glu384, Tyr523, and Arg522) via hydrogen bonds. It also bound to the sites of ACE (Glu162, Asp377, and Glu376) via salt bridges. Additionally, RLPHV bound to the sites of ACE (Val379, Val380, His383, His387, Val518, and Phe527) through hydrophobic interaction. TVKPGL bound to the sites of ACE (Gln281, Thr282, Ala354, Ala356, Glu411, and His513) using hydrogen bonds. It also bound to the sites of ACE (Glu162 and Lys511) via salt bridges. Moreover, TVKPGL bound to the sites of ACE (His353, Val380, His383, Phe512, and Tyr523) via hydrophobic interaction. KALVAP bound to the sites of ACE (Ala354, His353, and His383) using hydrogen bonds. It also bound to the sites of ACE (Glu376, Asp415, and Arg522) using salt bridges. Furthermore, KALVAP bound to the sites of ACE (Val380, His387, and Val518) via hydrophobic interaction. All three peptides formed coordination bonding with the Zn^2+^ ion of ACE.

Pina and Roque [36] reported that the main interaction residues of the ACE active site contained three active pockets (S1, S2, and S1′); the S_1_ pocket included the Tyr523, Glu384, and Ala354 residues. The Tyr520, Lys511, His353, His513 and Gln281 residues corresponded to the S2 pocket, and S1′ contained Glu162 residues and the HEXXH zinc-binding motif (His383, His387 and Glu411) [37]. RLPHV bound to four residues of the S1 and S2 pockets and bound to residues his383 and his387 of the HEXXH zinc-binding motif. TVKPGL bound to seven residues of the S1, S2, and S1′ pockets and bound to residues his383 and glu411 of the HEXXH zinc-binding motif. KALVAP bound to two residues in the S1 and S2 pockets, and it bound to the residues his383 and his387 in the HEXXH zinc-binding motif. However, the structures of RLPHV, TVKPGL, and KALVAP are different. 

Zn^2+^ ions are essential for maintaining ACE activity by binding to specific residues in the zinc-binding motif and forming tetrahedral coordinates [38]. Previous studies have shown that interactions between ACE-inhibitory peptides and the tetrahedral coordination of zinc ions can inhibit ACE activity [11,39,40]. Thus, interactions with zinc ions can effectively hinder the inhibitory activity of ACE. These three peptides can interact with the active site and Zn^2+^ ion of ACE, and the binding energy is relatively low, which may be the reason for the strong inhibitory activity of these three peptides.

The three peptides contain hydrophobic amino acids V, L, and P at the C-terminus. The short-chain peptides containing hydrophobic amino acids at the C-terminus displayed considerable potency as ACE-inhibitory peptides [41].

### 2.7. Stability of Peptides after Simulated Digestion In Vitro

The activity of peptides changes after gastrointestinal digestion. On this basis, the stability of the in vitro simulated gastrointestinal digestion end products of the peptides was determined using simulated gastrointestinal digestion. The results showed that the stabilities of the RLPHV, TVKPGL, and KALVAP peptides were 82%, 90%, and 78% (Table 3), respectively. The final content of these peptides was above 70%, which might be related to the structure of the peptides. Peptides containing proline have stronger resistance to gastrointestinal digestion [42]. These three peptides all contained proline to avoid degradation. In addition, the difficulty of peptide digestion and their hydrophobicity also had a certain dependence [43,44]. In general, the strong resistance of the three peptides to gastrointestinal digestion contributed to their subsequent development and utilization.

## 3. Materials and Methods

### 3.1. Materials

*Zizyphus jujuba* was purchased from a local market (Ningbo, China). All the peptides were synthesized (purity > 98%) by Chutai Biological Engineering Co., Ltd. (Hangzhou, China). Hippuryl-histidyl-leucine (HHL) and ACE were obtained from Sigma-Aldrich (St. Louis, MO, USA). The RP-HPLC chemicals and reagents were HPLC grade, and they were purchased from Merck Drugs & Biotechnology Ltd. (Beijing, China). All other reagents were commercially available and analytical grade.

### 3.2. Preparation of Zizyphus jujuba Protein

*Zizyphus jujuba* proteins were prepared according to the method of Memarpoor-Yazdi, Mahaki, and Zare-Zardini [10] with minor modifications. They were peeled, nucleated after homogenization, and then cooked under high pressure at the ratio of 1:10 (jujuba:water). The condition of enzymatic hydrolysis was 1% cellulase and hemicellulase at pH 5 and 55 °C for 3 h. Subsequently, the enzyme was inactivated (95 °C for 15 min) after enzymatic hydrolysis with 1% pectinase at pH 3.5 at 50 °C for 3 h. After centrifugation at 3000 rpm, the *Zizyphus jujuba* proteins were obtained via alkali-soluble acid precipitation. Finally, they were vacuum freeze-dried and stored at −80 °C.

### 3.3. Prediction of the Activity of Zizyphus jujuba Proteins Based on In Silico Digestion

The main protein components were identified using proteomics of *Zizyphus jujuba* proteins. The top 15 proteins with the strongest intensity identified in *Zizyphus jujuba* proteins were used to predict the peptides after digestion using enzymatic hydrolysis following the order of pepsin, trypsin, and chymotrypsin (https://biochemia.uwm.edu.pl/biopep-uwm/, accessed on 12 April 2020). 

### 3.4. Peptidomics Analysis of Zizyphus jujuba Proteins after In Vivo Digestion

Healthy C57BL/6 mice (female, 9 weeks old, 20.1 ± 0.3 g) were purchased from Shanghai SLAC Laboratory Animal Co., Ltd. (Shanghai, China). The mice were fasted overnight before the first experiment. The mice were divided into the blank and *Zizyphus jujuba* protein groups, with 3 mice in each group. The *Zizyphus jujuba* protein group was given 200 mg/kg by gavage. After 60 min, the mice were sacrificed, and their small intestines were taken out and placed in a Petri dish with normal saline. The outer wall of the small intestine was washed lightly and injected with 1 mL of saline, and then it was divided into multiple sections. The intestinal juice and the contents were squeezed gently and put into a 2 mL centrifuge tube, were centrifuged at 12,000 r/min for 20 min at 4 °C, and were freeze-dried, and underwent desalination analysis.

The peptide of *Zizyphus jujuba* was desalted using a C_18_ column and was freeze-dried, and its sequence was identified via ultra-high-performance liquid chromatography (UPLC) coupled with an Orbitrap Exactive mass spectrometer (Thermo Fisher Scientific, Waltham, MA, USA). All mass spectrometer parameters were reported in Wen et al. [45]. The sample was dissolved with 5 μL of 10 μL eluent A (0.1% formic acid aqueous solution), and the peptide was captured on a PepMap C_18_ column (100 μm × 2 cm) at flow rate of 10 μL/min for 3 min. The peptides were then separated via gradient elution chromatography on the PepMap C_18_ column (75 μm × 25 cm). The separation gradient for eluent B (acetonitrile solution of 0.1% formic acid) was from 5% to 30% within 60 min. The flow rate was 200 nL/min and the column temperature was 55 °C. The data dependence mode was adopted to switch automatically between MS and MS/MS. The full scan settings were as follows: automatic gain control target of 5 × 10^5^, resolution of 70,000, scan range of 100–1600 *m*/*z*, and maximum injection time of 50 ms. The fifteen most intense precursor ions were used for high-energy collisional dissociation. The error tolerance of parent mass and fragment mass was set at 10.0 ppm and 0.1 Da, respectively, without any enzyme.

The original data were analyzed using PEAKS software (PEAKS Online X build 1.7.2022-05-03_094023, Bioinformatics Solutions, Waterloo, ON, Canada). PEAKS can perform de novo sequencing and protein identification (PEAKS DB). The parameters of ab initio sequencing and database retrieval were as follows: NCBI *Zizyphus jujuba* database (https://www.ncbi.nlm.nih.gov/protein/?term=Zizyphus+jujuba, accessed on 12 May 2021); non-enzymatic digestion, the tolerance of primary mass spectrometry was 10 ppm; the tolerance of secondary mass spectrometry was 0.02 Da, no fixed modification; the variable modification was set to methionine oxidation; N-terminal acetylation, and charges were +1, +2, +3, and +4. The false positive rate (FDR) of peptide identification was 1%. For de novo sequencing, results of average local confidence (ALC) and data confidence greater than 80% and 95% were more credible, respectively.

### 3.5. 3D-QSAR Model Setting and Screening for ACEI Peptides

The ACEI peptides used were from our group and the DFBP database (http://www.cqudfbp.net/index.jsp, accessed on 21 June 2021). The peptides were collected according to the length and activity to ensure the length of each peptide had a wide range of activities. To avoid errors between different laboratories, we used a large number of ACEI peptides measured and a similar method for the determination of activity. A total of 46 ACEI peptides were collected and used; 34 peptides were used as the training set (Table S2), and 12 peptides were used as the test set (Table S2).

Discovery Studio (DS 2020) was used to set up the 3D-QSAR model. The method was designed according to the method reported by Yan et al. [17] with minor modifications. The information in the database is demonstrated in Table S2. Molecular alignment was performed using Molecular Overlay dialog to align to the hexapeptide Leu-Val-Leu-Pro-Gly-Glu, which was found and reported in our previous studies, to obtain an accurate and reliable 3D QSAR model.

### 3.6. ACE-Inhibitory Activity Assay In Vitro

The method used to determine ACE-inhibitory activity was designed according to that of Sangsawad et al. [46] with minor modifications. Peptides were dissolved in distilled water at a concentration of 1 mg/mL. The reaction mixture comprised 50 μL of peptide and 50 μL of ACE (25 mU/mL), undergoing pre-incubation for 5 min at 37 °C. Subsequently, 150 μL of substrate (8.3 mM HHL in 0.1 M Tris-HCl buffer with 500 mM NaCl at pH 8.3) was introduced, and the sample was further incubated at 37 °C for 60 min. Following the completion of the reaction, it was terminated via the addition of 1 M HCl. The contents of hippuric acid (HA) could be quantified through C_18_ column of Agilent 1260 HPLC. The elution was performed with 70% mobile phase A (pure water with 1% TFA) and 30% mobile phase B (ACN with 0.1% TFA) for 10 min, the retention time of hippuric acid was 4.1 min, and there were HHL peaks at 6.5 min. IC_50_ was used to express the activity of hydrolysate; all analyses were carried out under five different concentrations in triplicate. The calculation formula of the inhibition rate was as follows:

$$\mathrm{Inhibition}\;\mathrm{activity}\;(\%)=\frac{{\mathrm{HA}}_{\mathrm{control}}-{\mathrm{HA}}_{\mathrm{sample}}}{{\mathrm{HA}}_{\mathrm{control}}-{\mathrm{HA}}_{\mathrm{blank}}}\times 100\%$$

where HA_control_, HA_sample_, and HA_blank_ express the relative areas of the HA peak in the control samples, digestion samples, and blank samples, respectively.

### 3.7. Binding of Peptides to ACE via Molecular Docking 

The molecular docking method was designed according to that of Pekkoh et al. [47] with minor modifications. ACE structure (PDB ID: 1o86) was derived from the RCSB PDB Protein Data Bank (https://www1.rcsb.org/, accessed on 25 April 2021). Before docking, water molecules were removed, whereas the cofactors of zinc and chloride atoms were retained in the model. The structure of peptides was constructed using ChemDraw (CD, version 18.1, Cambridge software). Docking was run using Discovery Studio 2020 with a radius of 27.00 Å, including x coordinates of 35.0312, y coordinates of 70.1300, and z coordinates of 77.4545 for ACE.

### 3.8. Stability In Vitro Digestion 

The stability in vitro digestion followed the method we reported previously with minor modifications [48]. Pepsin (1000 U/mL) and pancreatin (50 U/mL) were used to digest the peptide (0.5 mg/mL). A Q Exactive system (Thermo Fisher Scientific, USA) equipped with ESI in positive ion mode was used for mass spectrometric detection.

$$Stability\;\%=\frac{Peak\;area\;after\;digestion\;sample}{Peak\;area\;before\;digestion\;sample}\times 100\%$$

### 3.9. Statistical Analysis

All the data were subjected to Statistical Analysis software, version 9.0 (SAS Institute, Cary, NC, USA), and were used to evaluate the differences in mean values of the data using one-way analysis of variance (ANOVA) and Duncan’s multiple range test. Values of *p* less than 0.05 were considered statistically significant.

## 4. Conclusions

After in silico simulated gastrointestinal digestion, the potential activities of the peptides derived from the *Zizyphus jujuba* proteins were predicted using the BIOPEP-UWM™ database. Subsequently, the actual peptides present in the intestines of the mice after the oral administration of the *Zizyphus jujuba* proteins were collected and analyzed. Through peptidomics and a 3D-QSAR model analysis, three novel active peptides (RLPHV, TVKPGL, and KALVAP) in *Zizyphus jujuba* were discovered. These peptides displayed ACE IC50 values of 3.81, 6.01, and 17.06 μM, respectively. It was revealed that these peptides exert their activity by interacting with the hydrogen bonds within the ACE active pockets of S1, S_1_′, and S_2_′. While the metabolism of peptides outside the gastrointestinal tract is indeed crucial, it is worth mentioning that this screening method based on gastrointestinal digestion is a significant and meaningful attempt, although many peptides have a plasma half-life of just minutes. This study could provide valuable insights into developing oral peptide drugs and functional foods.

## Figures and Tables

**Table 1:** ACE-inhibitory activity of jujube proteins after digestion.

Sample Name	ACE-Inhibitory IC_50_ (mg/mL)
Simulate gastrointestinal digestion of jujube protein	0.34
Jujube protein hydrolysates in mice intestinal tract	0.21

**Table 2:** Screening of the ACE-inhibitory peptides using the 3D-QSAR model.

No	Sequence	Peptide	Exact Mass	ACE Inhibition Rate (%) (2 mg/mL)	ACE IC_50_ (µM)
1	Leu-Glu-Lys-Pro-Leu-Leu	LEKPLL	711.45	24.2	________
2	Leu-Glu-Lys-Leu-Val-Thr	LEKLVT	701.43	30.5	________
3	Arg-Leu-Pro-His-Val	RLPHV	620.38	100.0	6.01
4	Thr-Val-Lys-Pro-Gly-Leu	TVKPGL	613.38	100.0	3.81
5	Tyr-Leu-His-Leu	YLHL	544.30	65.4	________
6	Arg-Phe-Pro-Arg	RFPR	574.33	22.9	________
7	Lys-Ala-Leu-Val-Ala-Pro	KALVAP	597.38	100.0	17.06
8	Lys-Val-Lys-Pro-Leu	KVKPL	583.41	−14.2	________
9	Pro-Arg-Pro-Lys-Pro-Pro-Pro	PRPKPPP	787.47	74.8	________
10	Pro-Glu-Arg-Lys	PERK	528.30	4.5	________
	* Positive control	Captopril	5 ng/mL	63.4	

* The ACE IC_50_ of Captopril ranges from 0.00179 to 0.025 μM (equivalent to 0.389–5.43 ng/mL) [34,35].

**Table 3:** CDOCKER energy of three ACEI peptides.

Name	CDOCKER Energy (kcal/mol)	CDOCKER Interaction Energy (kcal/mol)	Simulated Gastrointestinal Digestion Stability %
RLPHV	−106.075	−101.716	82
TVKPGL	−104.036	−90.9375	90
KALVAP	−102.312	−110.661	78

## Data Availability

No new data were created or analyzed in this study. Data sharing is not applicable to this article.

## Supplementary Materials

The following supporting information can be downloaded at: https://www.mdpi.com/article/10.3390/ijms242115848/s1.
