# Supplementary material for: Screening of Oral Potential Angiotensin-Converting Enzyme Inhibitory Peptides from Zizyphus jujuba Proteins Based on Gastrointestinal Digestion In Vivo
Source: Int J Mol Sci. 2023 Oct 31;24(21):15848. doi: 10.3390/ijms242115848 (PMC10648141; doi:10.3390/ijms242115848)
Supplement: Supplementary file 1 [file ijms-24-15848-s001.zip › ijms-2639409-supplementary.pdf]

Article

Screening of Oral Potential Angiotensin-Converting Enzyme Inhibitory Peptides from *Zizyphus jujuba* Proteins Based on Gastrointestinal Digestion In Vivo

Xinchang Gao [1,2], Chaoying Zhang [3], Ning Wang [1], Jin-Ming Lin [2], Yali Dang [4,*] and Yufen Zhao [1,2,*]

[1] Institute of Drug Discovery Technology, Ningbo University, Ningbo 315211, China; gxch43@126.com (X.G.); wangning2@nbu.edu.cn (N.W.)
[2] Department of Chemistry, Tsinghua University, Beijing 100084, China; jmlin@mail.tsinghua.edu.cn
[3] Chinese Academy of Fishery Sciences, Beijing 100141, China; cyzhang0317@cafs.ac.cn
[4] College of Food and Pharmaceutical Sciences, Ningbo University, Ningbo 315211, China
* Correspondence: dangyali@nbu.edu.cn (Y.D.); yfzhao@tsinghua.edu.cn (Y.Z.)

Table S1. Bioactivity peptides of *Zizyphus jujuba* protein after silicon gastrointestinal digestion.

No.	Uniprot ID	Description	Dipeptidyl peptidase IV inhibitor	ACE inhibitor	Antioxidative peptide	Glucose uptake stimulating peptide	Renin inhibitor	DPP-III inhibitor
1	A0A6P3ZWP5	(R)-mandelonitrile lyase 3-like [Ziziphus jujuba]	23	19	2	3	2	2
2	A0A6P3ZAQ7	basic 7S globulin-like [Ziziphus jujuba]	32	12	0	4	3	5
3	A0A6P3ZF39	heat shock cognate 70 kDa protein 2-like [Ziziphus jujuba]	20	13	3	2	2	1
4	A0A6P4BEQ2	probable mediator of RNA polymerase II transcription subunit 37e [Ziziphus jujuba]	23	15	3	0	1	2
5	A0A6P4A5H0	chaperone protein ClpB1 [Ziziphus jujuba]	33	37	6	4	1	1
6	A0A6P6GI53	alpha-xylosidase 1 [Ziziphus jujuba]	59	39	3	4	3	6

7	A0A6P4A0H8	primary amine oxidase-like [Ziziphus jujuba]	50	34	4	4	6	9
8	A0A6P4A9Y3	beta-galactosidase 8 isoform X1 [Ziziphus jujuba]	51	36	2	1	2	2
9	A0A6P4ATG6	GDSL esterase/lipase 1-like [Ziziphus jujuba]	24	19	0	0	2	5
10	A0A6P4ACD1	V-type proton ATPase catalytic subunit A [Ziziphus jujuba]	29	18	3	1	3	1
11	A0A6P4AUL2	alpha-mannosidase-like [Ziziphus jujuba]	60	45	8	3	4	6
12	R4L6S9	heat shock protein 70 [Ziziphus jujuba]	20	13	4	2	1	1
13	A0A6P3Z888	heat shock cognate 70 kDa protein-like [Ziziphus jujuba]	25	13	3	2	1	2
14	A0A6P4B4C0	V-type proton ATPase subunit B 2 isoform X1 [Ziziphus jujuba]	18	17	4	2	2	1
15	A0A6P4AF39	ATP synthase subunit beta, mitochondrial [Ziziphus jujuba]	16	18	4	4	1	2
Total			483	348	49	36	34	46

Table S2. Data of the 3D-QSAR model.

No	Sequences	PIC50	Predicted PIC50	Error
1	Leu-Pro-Gly-Val-Leu-Pro-Val-Ala*	0.11	-1.41	1.52
2	Thr-Tyr-Leu-Pro-Val-His*	-0.14	-1.35	1.21
3	Pro-Thr-His-Ile-Lys-Trp-Gly-Asp	-0.18	0.04	-0.22
4	Phe-Gln-Lys-Val-Val-Ala-Lys*	-0.32	-1.47	1.15
5	Thr-Phe-Gln-Gly-Pro-Pro-His-Gly-Ile-Gln-Val-Glu-Arg	-0.53	-0.66	0.13
6	Tyr-Glu-Thr-Gly-Asn-Gly-Ile-Lys	-0.57	-0.53	-0.04
7	Leu-Gly-Pro-Leu-Gly-His-Gln	-0.63	-0.42	-0.21
8	Ile-Ile-Ala-Pro-Pro-Glu-Arg	-0.94	-1.04	0.10
9	Cys-Cys-Asn-Lys	-0.95	-1.01	0.06
10	Ser-Leu-Pro-Gln-Asn	-0.98	-0.82	-0.16
11	Cys-Cys	-0.99	-1.39	0.40
12	Leu-His-Pro-Gly-Asp-Ala-Gln-Arg	-1.01	-0.98	-0.03
13	Leu-Val-Leu	-1.09	-1.34	0.25
14	Leu-Val-Leu-Pro-Gly-Glu	-1.13	-1.32	0.19
15	Cys-Cys-Asn-Lys-Ser-Val*	-1.18	-1.66	0.48
16	Asn-Ile-Phe-Tyr-Cys-Pro	-1.18	-1.25	0.07
17	Ala-His-Ser-Val-Arg-Phe-Tyr*	-1.21	-1.52	0.31
18	Leu-Phe-Arg-Gln*	-1.23	-1.78	0.55
19	Val-His-Leu-Pro-Pro*	-1.26	-1.67	0.41
20	Ala-His-Ser-Val-Arg-Phe*	-1.26	-1.49	0.23
21	Gly-Lys-Asp-Ala-Val-Ile-Val	-1.26	-1.39	0.13
22	Pro-Ala-Asn-Ile-Lys-Trp-Gly-Asp	-1.32	-1.26	-0.06
23	Tyr-Pro-Gly	-1.37	-1.64	0.27
24	Gly-Tyr-Ala-Leu-Pro-His-Ala	-1.44	-1.16	-0.28
25	His-Cys-His-Thr-Asn*	-1.48	-1.71	0.23
26	Phe-Cys-Leu-Tyr-Glu-Leu-Ala-Arg	-1.50	-1.45	-0.05
27	Leu-Gln-Lys-Trp*	-1.54	-1.81	0.27
28	Val-Leu-Pro-Tyr-Pro	-1.56	-1.42	-0.14
29	Asp-Phe-Gly	-1.65	-1.66	0.01
30	Leu-Pro-Gln-Asn-Ile-Leu-Pro*	-1.66	-1.95	0.29
31	Leu-Ala-Lys*	-1.68	-1.79	0.11
32	Gly-Arg-Val-Ser-Asn-Cys-Ala-Ala	-1.76	-1.75	-0.01
33	Asn-Met-Ala-Ile-Asn-Pro-Ser-Lys	-1.78	-1.58	-0.20
34	Pro-Ser-Phe-Gln-Pro	-1.86	-1.96	0.10
35	Gly-Ile-Pro-Leu-Pro-Leu-Ile	-1.87	-2.19	0.32
36	Tyr-Thr-Val-Phe	-1.94	-2.20	0.26
37	Lys-Val-Arg-Glu-Gly-Thr-Thr-Tyr	-2.01	-1.85	-0.16
38	His-Cys-His-Thr	-2.06	-2.18	0.12
39	Phe-Pro-Glu-Val-Phe-Gly-Lys	-2.15	-2.19	0.04
40	Gln-Val-Gly-Pro	-2.21	-2.22	0.01
41	Leu-Val-Leu-Pro-Gly-Glu-Leu-Ala-Lys	-2.26	-2.43	0.17
42	Ile-Gly-Pro-Gly-Leu-Gly-Arg	-2.32	-2.19	-0.13
43	Ala-Asp-Gly-Leu-Trp-Leu	-2.45	-2.21	-0.24
44	Phe-Pro-Gln-Tyr	-2.48	-2.38	-0.10
45	Lys-Ser-Val	-3.01	-2.81	-0.20
46	Ala-His-Ser-Val	-3.26	-2.83	-0.43

* Test set peptides (the external validation).

Table S3. The actual active Zizyphus jujuba peptides in mice intestine.

No	Sequence	Lenth	ALC (%)	RT	Area	Mass
1	Val-Val-Lys-Val-Lys-Val-Pro	7	93.1	40.8	1.72E+06	767.5269
2	Pro-Arg-Pro-Lys-Pro-Pro-Pro	7	87.5	38.6	7.42E+06	787.4705
3	His-Leu-Lys-Pro-Asp-Gln	6	97.9	33.9	2.24E+06	736.3868
4	Leu-Glu-Lys-Pro-Leu-Leu	6	97.4	46.8	3.10E+06	711.4531
5	Thr-Val-Lys-Pro-Gly-Leu	6	87.8	36.3	7.18E+06	613.3799
6	Glu-Leu-Tyr-Leu-Ala-Gly	6	87.4	39.0	7.05E+07	664.3431
7	Leu-Asp-Phe-Ser-Gly-Ser	6	87.3	24.5	6.53E+06	624.2755
8	Leu-Leu-Ala-Ala-Ala-Tyr	6	85.4	28.6	5.49E+06	620.3533
9	Lys-Ala-Leu-Val-Ala-Pro	6	85.1	36.1	1.24E+06	597.3849
10	Tyr-Gly-Gly-Gly-Gly-Pro	6	84.8	8.7	1.05E+06	506.2125
11	Ser-Gly-Leu-Leu-Gly-Lys	6	83.3	57.1	3.45E+06	573.3486
12	Glu-Tyr-Leu-Leu-Ala-Gly	6	83.1	42.4	3.06E+07	664.3431
13	Leu-Glu-Lys-Leu-Val-Thr	6	82.5	36.9	2.27E+06	701.4323
14	Glu-Phe-Thr-Leu-Val-Gly	6	82.4	38.5	2.83E+07	664.3431
15	His-Leu-Val-Val-His	5	98.6	37.1	2.13E+06	603.3492
16	Arg-Val-Pro-Pro-Arg	5	98	36.7	1.80E+07	623.3867
17	His-Leu-Arg-Pro-Asn	5	95.8	34.9	3.09E+06	635.3503
18	Arg-Leu-Pro-His-Val	5	93.9	44.6	4.92E+05	620.3758
19	Lys-Leu-His-Pro-Val	5	93.3	43.3	4.12E+06	592.3696
20	His-Pro-Pro-Pro-Pro	5	93.2	25.5	1.09E+07	543.2805
21	His-Leu-Lys-Pro-Val	5	92.8	43.3	4.12E+06	592.3696
22	Gln-Ala-Pro-Ser-Val	5	92.4	12.7	2.94E+06	500.2595
23	Lys-Val-Arg-Pro-Leu	5	92.1	40.3	2.09E+06	611.4119
24	Leu-Pro-Lys-Glu-Pro	5	88.9	27.8	8.51E+06	582.3377
25	Lys-Val-Leu-Gln-Pro	5	87.6	30.4	1.17E+07	583.3693
26	Lys-Val-Lys-Pro-Leu	5	86.1	46.3	2.27E+06	583.4057
27	Glu-Pro-Val-Leu-Lys	5	86	34.0	1.10E+06	584.3533
28	Ser-Leu-Leu-Gly-Lys	5	85.8	54.6	1.46E+07	516.3271
29	Glu-Gly-Gly-Gly-Lys	5	85.7	26.4	9.55E+06	446.2125
30	Val-Asn-Leu-Asp-Thr	5	85.2	13.0	5.65E+06	560.2806
31	Asp-Gly-Phe-Ser-Leu	5	85.1	37.0	3.43E+06	537.2435
32	Gln-Val-Leu-Pro-Lys	5	84.9	37.3	6.57E+05	583.3693
33	Asp-Gly-Ser-Ser-Phe	5	84.8	15.8	3.28E+06	511.1914
34	Gln-Gly-Ala-Pro-Arg	5	84.7	37.2	4.84E+06	527.2816
35	Ser-Phe-Asp-Ser-Thr	5	83.5	13.5	3.71E+06	555.2177

36	Ser-Gly-Val-Gly-Pro	5	83.1	11.0	4.38E+06	415.2067
37	Leu-Leu-Arg-Pro-His	5	82.5	39.1	5.08E+05	634.3915
38	His-Val-Arg-Glu-Pro	5	82.2	34.8	3.52E+06	636.3344
39	Ala-Gly-Lys-Leu-Arg	5	81.7	33.1	6.07E+06	543.3492
40	Asn-Lys-Arg-Thr-Pro	5	81.3	32.6	4.76E+06	614.35
41	His-Leu-Arg-Asn-Pro	5	93.2	34.9	3.09E+06	635.3503
42	Tyr-Leu-His-Leu	4	97.8	43.9	1.89E+06	544.3009
43	Pro-Pro-His-Leu	4	97.5	36.1	6.44E+06	462.2591
44	His-Arg-Pro-Leu	4	97.2	38.2	1.27E+07	521.3074
45	Phe-Leu-His-Leu	4	97	48.9	1.03E+06	528.306
46	Arg-Leu-Ala-Lys	4	96.7	38.3	1.71E+07	486.3278
47	Arg-Val-His-Leu	4	96.3	46.8	2.55E+06	523.3231
48	His-Leu-Pro-Pro	4	96	35.6	4.46E+06	462.2591
49	Glu-Met-Pro-Arg	4	95.6	37.7	5.42E+06	531.2475
50	Lys-Leu-His-Asn	4	95.5	27.5	1.05E+07	510.2914
51	Trp-Lys-Pro-Arg	4	95.4	44.2	6.69E+06	585.3387
52	Arg-Val-Val-Lys	4	95.4	38.2	8.45E+06	500.3434
53	Leu-Val-Lys-Leu	4	95.2	40.7	1.15E+06	471.342
54	His-Leu-His-Val	4	95.2	42.1	1.45E+06	504.2809
55	Leu-Leu-Lys-His	4	94.8	34.8	2.35E+06	509.3326
56	Lys-Val-Asp-Leu	4	93.9	36.4	1.02E+07	473.2849
57	Gln-Arg-Pro-Lys	4	93.7	29.1	4.73E+06	527.318
58	Arg-Phe-Pro-Arg	4	93.5	39.0	2.17E+06	574.3339
59	Glu-Arg-Leu-Gln	4	93.5	33.4	4.78E+05	544.2969
60	Arg-Val-His-Pro	4	93.1	38.7	1.53E+08	507.2917
61	Leu-Lys-His-Pro	4	93	32.8	2.78E+06	493.3012
62	Thr-Arg-Leu-Arg	4	92.9	39.4	5.07E+05	544.3445
63	Glu-Val-Leu-His	4	92.4	40.5	8.06E+04	496.2645
64	Arg-Gln-His-Leu	4	91.9	39.7	2.66E+06	552.3132
65	Leu-Val-Arg-Lys	4	91.9	32.9	3.27E+06	514.3591
66	Phe-Ser-Phe-Asp	4	91.9	29.1	6.64E+07	514.2064
67	Lys-Ser-Lys-Arg	4	91.8	42.8	1.84E+06	517.3336
68	Leu-Arg-His-Pro	4	91.7	33.7	1.34E+07	521.3074
69	Leu-Pro-Lys-Leu	4	91.7	34.2	9.55E+05	469.3264
70	Lys-Val-Thr-His	4	91.7	30.7	1.55E+06	483.2805
71	Leu-Arg-Phe-Gln	4	91	39.1	1.56E+07	562.3227
72	Pro-Leu-Lys-Pro	4	90.9	43.3	1.00E+06	453.2951

73	Lys-Leu-Val-His	4	90.9	36.6	1.10E+07	495.3169
74	Ser-Arg-Lys-Leu	4	90.6	41.3	9.23E+06	502.3227
75	Phe-Val-Arg-Pro	4	90.6	33.5	3.33E+05	517.3013
76	Lys-Met-Lys-Phe	4	90.2	50.7	2.03E+06	552.3094
77	Leu-Gln-His-Leu	4	90.1	33.2	3.89E+06	509.2962
78	His-Arg-Pro-Glu	4	90	33.8	3.26E+07	537.2659
79	Val-Gln-Leu-Lys	4	89.7	38.0	3.17E+06	486.3166
80	Ala-Leu-His-Arg	4	89.5	34.1	4.35E+06	495.2917
81	Asn-Lys-Lys-Leu	4	89.1	34.0	7.93E+06	501.3275
82	Lys-Lys-Met-Ala	4	89	39.7	5.41E+06	476.2781
83	Arg-Leu-His-Leu	4	88.7	46.3	4.81E+06	537.3387
84	His-His-Phe-Pro	4	88.4	43.7	9.95E+05	536.2496
85	Leu-Pro-Lys-Pro	4	88.2	38.5	3.96E+06	453.2951
86	Glu-Pro-Lys-His	4	88.2	32.4	4.64E+06	509.2598
87	Leu-Val-Glu-Lys	4	87.8	21.9	1.08E+06	487.3006
88	Val-Glu-Arg-Leu	4	87.2	33.1	1.32E+06	515.3067
89	Pro-Asp-Arg-His	4	87	33.7	5.30E+06	523.2503
90	Val-Asn-Leu-Ala	4	86.5	12.2	9.64E+07	415.2431
91	Arg-Gln-Leu-Lys	4	86.1	33.1	6.07E+06	543.3492
92	Arg-Thr-Pro-Leu	4	85.8	34.3	2.09E+06	485.2962
93	Lys-Leu-Gln-Leu	4	85.7	41.1	2.81E+06	500.3322
94	Pro-Glu-Arg-Lys	4	85.5	34.0	2.31E+06	528.302
95	Glu-Ser-Phe-Glu	4	85.3	13.4	1.75E+06	510.1962
96	Asp-Ser-Ser-Phe	4	85.2	17.7	1.27E+06	454.17
97	Thr-Pro-Pro-Arg	4	84.8	8.4	2.77E+05	469.2649
98	Asn-Lys-His-Leu	4	84.8	34.7	3.23E+07	510.2914
99	Lys-Ala-Phe-Phe	4	84.3	33.3	8.02E+05	511.2794
100	Leu-Arg-Pro-Arg	4	84.2	37.8	9.17E+06	540.3496
101	Lys-Leu-Asn-His	4	84	32.4	2.91E+07	510.2914
102	Leu-Ser-Phe-Asp	4	83.5	22.0	9.34E+06	480.222
103	Val-Lys-Val-Arg	4	82.3	39.2	1.18E+06	500.3434
104	Ser-Asp-Phe-Val	4	81.7	22.3	2.35E+06	466.2064
105	His-Gly-Leu-Pro	4	81.7	32.6	1.11E+07	422.2278
106	His-Ala-Leu-Pro	4	81.7	34.4	4.80E+06	436.2434
107	Leu-Lys-Ala-Pro	4	81.3	36.7	8.39E+06	427.2794
108	Asn-Leu-Gln-Lys	4	81	40.3	1.05E+06	501.2911
109	Phe-Gly-Arg-Pro	4	80.8	29.5	2.14E+06	475.2543

110	Val-Val-Val-Ala	4	80.6	15.3	3.67E+06	386.2529
111	His-Arg-Val	3	89	34.8	1.94E+06	410.239
112	Arg-Arg-Ser	3	85.5	31.6	1.96E+07	417.2448
113	Arg-Arg-Asn	3	85.3	30.8	1.70E+07	444.2557
